# Vanadium Nitride Nanoparticles Grown on Carbon Fiber Cloth as an Advanced Binder-Free Anode for the Storage of Sodium and Potassium Ions

**DOI:** 10.3390/ma16175820

**Published:** 2023-08-25

**Authors:** Yiwei Qin, Haimin Zhang, Jiachen Yanghe, Jing Yang, Wei Li, Xiaojun Zhao, Sainan Liu

**Affiliations:** 1 School of Materials Science and Engineering, Central South University, Changsha 410083, China; yiweiqin.csu@foxmail.com; 2 Hunan Zoomlion Neo Material Technology Co., Ltd., Changsha 410083, China; zhanghaimin@csu.edu.cn; 3 School of Minerals Processing and Bioengineering, Central South University, Changsha 410083, China; 8204211522@csu.edu.cn (J.Y.); 215612104@csu.edu.cn (J.Y.); 4 Powder Metallurgy Research Institute, Central South University, Changsha 410083, China; csuliw@csu.edu.cn

**Keywords:** vanadium nitride, anode, carbon fiber cloth, sodium-ion batteries, potassium-ion batteries

## Abstract

The escalating demand for sustainable and high-performance energy storage systems has led to the exploration of alternative battery technologies for lithium-ion batteries. Sodium-ion batteries (SIBs) and potassium-ion batteries (PIBs) have emerged as promising candidates because of their abundant Na/K resources, inexpensive costs, and similar chemistries to lithium-ion batteries. However, inherent challenges, such as large ionic radii, sluggish kinetics, and serious volume expansion, necessitate the development of robust and efficient anode materials for SIBs and PIBs. Vanadium nitride has attracted increasing attention as a viable anode due to its high electronic conductivity and potential capacity. In this study, we report on a flexible electrode for SIBs and PIBs that creates binder-free anodes by synthesizing vanadium nitride nanoparticles grown directly on carbon fiber cloths (VN/CFC). The unique architecture and binder-free nature of this anode ensure a robust electrode–electrolyte interface and enhance its electron/ion transport kinetics. The results demonstrate that the material exhibits an outstanding specific discharge capacity of 227 mAh g^−1^ after undergoing 1000 cycles at a current density of 2 A g^−1^ for SIBs. An electrochemical analysis indicated that the excellent performance of the material is attributed to the bind-free structure of carbon fiber cloth and the fast kinetics of surface pseudo-capacitive contribution. Furthermore, the material continues to demonstrate an impressive performance, even for PIBs, with a specific discharge capacity of 125 mAh g^−1^ after 1000 cycles at a current density of 1 A g^−1^. This study provides a new perspective for designing and developing advanced binder-free anodes for the storage of sodium and potassium ions, paving the way for high-performance energy storage applications.

## 1. Introduction

The rapid development of portable electronic devices and electric vehicles has increased demands for high-performance energy storage systems [1]. Among various energy storage technologies, lithium-ion batteries (LIBs) are the mainstream electrochemical energy storage containers because of their high energy density and long cycle life [2,3,4]. However, with the continuous expansion of market demands, the issues of limited lithium resources and rising costs are becoming increasingly prominent. This has prompted researchers to focus on possible alternative systems for lithium-ion batteries [5,6,7].

Sodium-ion batteries (SIBs) and potassium-ion batteries (PIBs) have emerged as promising candidates because of their similar working principles to LIBs, abundant resources, and inexpensive costs [8,9,10,11,12]. Yet, the practical applications of SIBs and PIBs are hindered by the large ionic radii of potassium and sodium ions, which lead to sluggish kinetics and serious volume expansion during the charge–discharge process [13]. The development of robust and efficient anode materials for SIBs and PIBs is, therefore, of critical importance. Carbonaceous materials, alloy reaction materials, and transition metal oxides/sulfides have been extensively researched in the literature due to their respective advantages [7]. Among these, some electrode materials have excellent Faraday quasi-capacitance and a wide electrochemical potential window, such as transition metal oxides [14,15,16], transition metal nitrides [17,18,19], and transition metal sulfides [20,21], which are promising anode materials for sodium-/potassium-ion batteries [22,23,24,25,26,27,28,29]. Vanadium nitride (VN), a typical transition metal nitride, belongs to a cubic crystal system with the space group Fm3m and a cell parameter of a = 4.13916 nm. It has attracted much attention due to its high theoretical capacity, good electronic conductivity, and excellent structural stability [30,31]. For example, Yuan [32] reported that VNQD@NC HSs, with nanocrystalline sizes that are significantly decreased compared to VN, and their distinctive hollow nanohybrids have excellent electrochemical properties. VN displayed a reversible capacity of 306 mAh g^−1^ over 1400 cycles at a current density of 1 A g^−1^. As previously reported, to further improve the performance of VN anodes, designing nanostructures, combining vanadium nitride with carbon-based materials, and doping have been identified as effective strategies [33,34]. 

Therefore, in this study, we report on a novel method to grow VN nanoparticles directly on carbon fiber cloths (VN/CFC) to create a binder-free anode for both SIBs and PIBs. Carbon fiber cloth is a commonly used flexible substrate with a high conductivity. The prepared composite material can be directly used as an electrode and then assembled into a battery, avoiding the use of adhesives and conductive agents. The binder-free nature of the VN/CFC anode not only ensures a robust electrode–electrolyte interface but also enhances the electron/ion transport kinetics. The VN/CFC anode exhibited impressive electrochemical performances for SIBs and PIBs, showing an outstanding specific discharge capacity of 227 mAh g^−1^ after undergoing 1000 cycles at a current density of 2 A g^−1^ for SIBs and a specific discharge capacity of 125 mAh g^−1^ after 1000 cycles at 1 A g^−1^ for PIBs. An electrochemical analysis indicated that its excellent performance is attributed to the bind-free structure of carbon fiber cloths and the fast kinetics of the pseudo-capacitive contribution of the surface. The results demonstrate the anode’s potential in high-performance energy storage applications. This study provides a new perspective for designing and developing advanced binder-free anodes for storing sodium and potassium ions.

## 2. Experimental Section

### 2.1. Synthesis of VN/Carbon Fiber Cloth and VN Powder

Firstly, the commercial carbon fiber cloth was oxidized to obtain the treated carbon fiber cloth for subsequent experiments. The detailed steps are as follows: cut 0.5 g of the commercial carbon fiber cloth and place it in the mixed solution of sulfuric acid (H_2_SO_4_, 95 wt.%, 20 mL) and nitric acid (HNO_3_, 65 wt.%, 10 mL). Keep stirring and slowly add 3 g of potassium permanganate (KMnO_4_) into the solution. The whole process was carried out in an ice-water bath so that the temperature of the mixed solution was lower than 10 °C, and the next step was carried out after 3 h. Then, 100 mL of deionized water was added to the solution, and the whole process was controlled at a temperature lower than 25 °C. After stirring for 6 h, hydrogen peroxide (H_2_O_2_, 30 wt.%, 10 mL) was added to the solution. The oxidation process lasted for 30 min. The treated carbon fiber cloth was taken out, washed repeatedly with deionized water and ethanol, and then dried in an oven at 60 °C for 12 h.

Then, the precursor grown on carbon fiber cloth was prepared. While stirring, 0.265 g of vanadyl acetylacetonate was added to 25 mL of isopropanol. Ultrasonic treatment was performed for 15 min, and then the stirring and ultrasonic treatment process was repeated again. The mixed solution was poured into a stainless-steel autoclave of 100 mL capacity, and a piece of the treated carbon fiber cloth was immersed in the reactor and maintained at 160 °C for 24 h. After naturally cooling down, the sample was repeatedly washed with deionized water and ethanol and then dried to obtain the carbon fiber cloth precursor. Finally, the carbon fiber cloth precursor was calcined in a NH_3_ atmosphere at 550 °C for 2 h to obtain the VN nanoparticles grown on the carbon fiber cloth (VN/CFC). During the preparation process, the powder obtained after hydrothermal treatment was mainly vanadium oxide, and VN was obtained after calcination in the NH_3_ atmosphere. The transformation that occurs during the preparation process is as follows:

C_10_H_14_O_5_ V → VxOy·nH_2_O → VN

For comparison, VN powder was also prepared without adding CFC. The solution without the addition of treated carbon fiber cloth was centrifuged after the hydrothermal process and dried overnight to obtain the powder precursor. The comparative sample was obtained under the same nitriding condition. 

### 2.2. Characterization Techniques

Scanning electron microscopy (JSM-7610FPlus, JEOL, Tokyo, Japan) was used to observe the morphologies of the prepared samples. Furthermore, the microstructure information was investigated via high-resolution transmission electron microscopy (HR-TEM, FEI Tecnai G2 F20 instrument, Stanford, CA, USA). The crystal structure of the prepared VN/CFC and VN samples was investigated via XRD, measured using TTR III X-ray diffraction with Cu Kα radiation (λ = 1.54178 Å, Rigaku, Tokyo, Japan) in the range of 5°~80°. Raman spectra (Renishaw InVia Qontor, London, UK) were used to determine the structural information of samples. The chemical composition of VN/CFC was determined using X-ray photoelectron spectroscopy (XPS) analysis via a Thermo Fisher Scientific ESCALAB Xi+ instrument (Carlsbad, CA, USA). 

### 2.3. Electrochemical Measurements

Flexible VN/CFC can be directly assembled as binder-free SIB and PIB anodes by cutting them into small pieces of 0.8 cm × 0.8 cm. In contrast, the VN sample was mixed with the binder polyvinylidene fluoride (PVDF) and the conductive agent (Super P) at a ratio of 8:1:1 in the preparation of the electrode, and the appropriate amount of NMP was added to obtain a slurry after mixing evenly. Then, the slurry was uniformly poured on a copper foil, dried in a vacuum oven at 80 °C for 10 h, and finally cut into small round pieces with a diameter of 12 mm.

We assembled the CR2016 coin cells used for testing in an argon glove box (O_2_ and H_2_O contents ≤ 0.1 ppm). The metallic Na and K foils were employed as the counter electrodes. The electrolyte of SIB is composed of 1 M NaPF6 dissolved in a 1:1 (volume) mixture of dimethyl carbonate (DMC)/ethylene carbonate (EC), adding 5 vol% fluorinated ethylene carbonates (FEC). Furthermore, a 0.8 M KPF_6_ dissolved in ethylene carbonate (EC) and diethyl carbonate (DEC) (taken at a 1:1 volume ratio) was employed as an electrolyte for PIBs. The GF/D glass fiber filter was used as a separator.

In the voltage range of 0.01~3 V, a CT2001A battery tester (manufactured by the LAND Electronic Co. Wuhan, China) was used to test the galvanostatic charge–discharge performances of the battery. Cyclic voltammetry (CV) at different sweep rates and electrochemical impedance spectroscopy (EIS) in the frequency range from 100 kHz to 0.01 kHz were employed in the CHI-600C workstation (CHI-600C, Shanghai, China). 

It involved establishing a correlation between the current dependence and the sweep rate (*v*), influenced by the charge–storage process. Equation (1) was used for this purpose, with a representing a constant and b representing the power-law exponent. *log (i)–log (v)* of the redox peaks (Equation (2)) was plotted to obtain the value of *b*, providing qualitative information about the kinetics of the charge storage mechanism [35].

(1) $$I=av^{b}$$

or

(2) logI=blogv+loga

when *b* is equal to 0.5, this indicates that the redox peak current is directly proportional to the square root of the sweep rate, suggesting a diffusion-controlled faradaic charge–storage process. On the other hand, as the *b* value approaches 1.0, it reveals a linear relationship between the peak current and the scan rate *v*, indicating a surface-controlled capacitor-like electrochemical response [36].

Equations (3) and (4) are as follows:

(3) $$i\;\left(V\right)=k_{1}v+k_{2}v^{1/2}$$

(4) $$i/v^{1/2}=k_{1}v^{1/2}+k_{2}$$

The current response at a fixed potential consisted of two distinct contributions: surface-capacitive and diffusion-controlled contributions. These two processes are described using constants *k*_1_ and *k*_2_, respectively.

## 3. Results and Discussion

**Figure 1:**
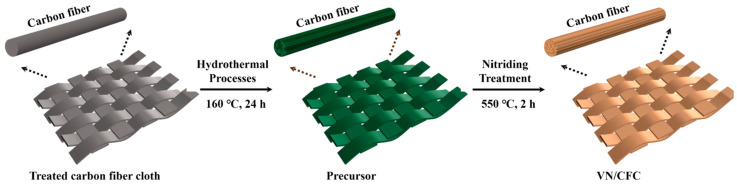
The preparation route of VN/CFC composite material.

The successful synthesis of VN/CFC composites is schematically illustrated in Figure 1. Here, vanadyl acetylacetonate serves as a source of vanadium. The treated carbon fiber cloth was used as a flexible substrate to obtain the carbon fiber cloth precursor. After the subsequent calcine process at 550 °C in NH_3_, the precursor was finally converted into the flexible 3D electrode material, VN/CFC.

From the SEM images, we can observe that there are a lot of impurities on the surface of a commercial carbon cloth (Figure S1a,b). After being treated with strong acid, the surface is significantly cleaner (Figure S1c,d). In addition, the surface of the treated carbon fiber has clearer folds, which may be due to the action of strong acids. The surface of the carbon fiber was oxidized, which deepened the depression of the surface. Simultaneously, a set of highly reactive groups, including hydroxyl and carboxylic groups, were incorporated into the material to render it hydrophilic. This modification facilitated the preparation conditions for the subsequent solvothermal loading of vanadium nitride.

**Figure 2:**
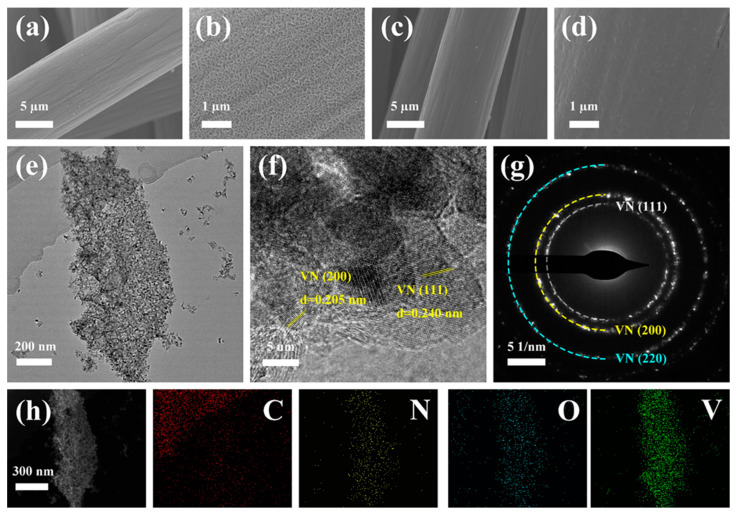
SEM micrographs of (**a**,**b**) carbon fiber cloth precursor, (**c**,**d**) VN/CFC, (**e**) TEM image, (**f**) HRTEM image, (**g**) SAED patterns, and (**h**) the corresponding element mappings obtained for the VN/CFC.

Vanadium oxide was uniformly grown on the surface of CFC with a nanoflower morphology after hydrothermal heating for 24 h (Figure 2a,b). The morphology of nanoflower disappeared after calcination, but the active material VN is well deposited over the CFC substrate without any notable signs of agglomeration and cracking (Figure 2c,d). This morphology facilitates an increase in the contact area of the electrolyte and a reduction in the volume expansion effect, which is of great significance for improving the electrochemical performance of SIBs and PIBs. The morphology (Figure S2a,b) of the VN powder precursor is consistent with that of the adherents on the surface of the carbon cloth precursor and consists of many small crystals agglomerated together. After nitriding, the petal-like morphology disappeared, but the state of particle aggregation did not change (Figure S2c,d). Elements V and N are also clearly visible in the mapping (Figure S2e), proving the existence of these two elements. The HRTEM image (Figure 2f) shows that the d-spacing value of the lattice fringe (0.240 and 0.205 nm) correspond to the (111) and (200) planes of VN, respectively. In addition, the HRTEM images (Figure 2f) show that the exfoliated samples are composed of multiple vanadium nitride crystal particles clustered together. Combined with the SEM image (Figure 2c,d), it can be judged that vanadium nitride particles grow on and completely cover the surface of the carbon fiber like bark. Figure 2g shows the selected area electron diffraction (SAED) patterns, which reveal several rings ascribed to the (111), (200), and (220) diffraction planes of VN. The energy-dispersive spectroscopy (EDS) elemental mapping images definitely indicate the co-existence of C, N, O, and V elements in the VN/CFC (Figure 2h).

**Figure 3:**
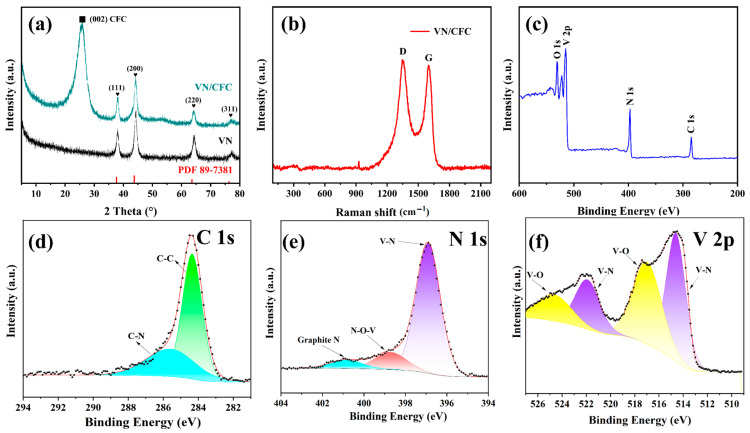
(**a**) XRD patterns of VN powder and VN/CFC, (**b**) Raman spectra of VN/CFC, (**c**) survey and high-resolution (**d**) C 1s (**e**) N 1s and (**f**) V 2p spectra recorded for the VN/CFC composite material.

The diffraction peaks of the VN powder and VN/CFC can be well indexed to JCPDS card No. 89-7381. However, unlike the VN powder, the VN/CFC pattern shows a broad peak at about 25.7°, which is attributed to the CFC. XRD peaks observed for both samples at 38.3°, 44.2°, 64.4°, and 77.2° are ascribed to the (111), (200), (220), and (311) planes of VN, respectively. No other peaks are visible (Figure 3a). The Raman spectra of VN/CFC are shown in Figure 3b. We can clearly observe the D-band at 1344.9 cm^−1^ and G-band at 1601.5 cm^−1^. The intensity ratio between the D-band and G-band is 1.054, confirming the amorphous characteristics of the as-prepared VN/CFC. The elemental compositions and valence states of VN/CFC were revealed using XPS, showing the existence of C, N, O, and V elements (Figure 3c).

The high-resolution C 1s spectrum revealed peaks at 284.8 and 285.5, which were assigned to C-C and C-N, respectively (see Figure 3d) [13]. The high-resolution N 1s spectrum of the VN/CFC contained three peaks at 397.0, 398.7, and 401.2 eV, which were associated with the V-N bond, V-O-N bond, and graphite N bond, respectively (see Figure 3e) [37]. The peaks at 530.5 and 531.8 agreed with the V-O and C-O bond configurations, respectively (see Figure S3). The high-resolution V 2p spectrum showed peak pairs at 514.7/522.1 and 517.0/524.8 eV, dominating with V-N and V-O bonds, respectively (see Figure 3f) [38]. These results indicate the successful synthesis of the VN/CFC.

**Figure 4:**
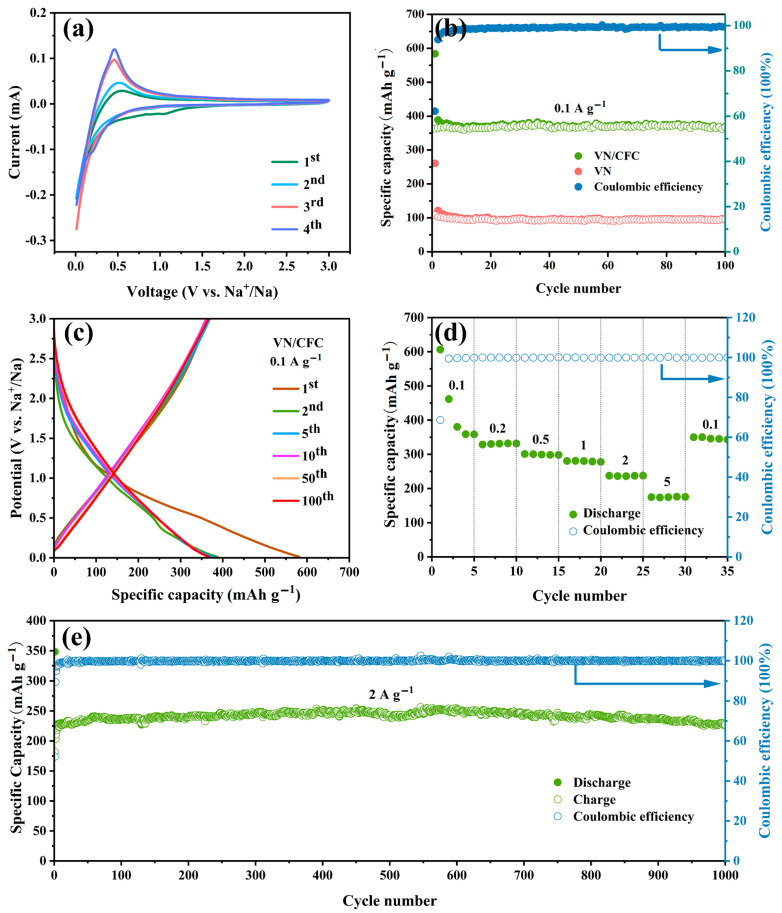
(**a**) CV curve (0.1 mV s^−1^) of VN/CFC-containing anode, (**b**) cycle performance of VN/CFC and VN powder anodes at 0.1 A g^−1^ current density, (**c**) galvanostatic charge–discharge curves of VN/CFC-based anode at 0.1 A g^−1^, (**d**) rate performances of VN/CFC SIBs, and (**e**) long-life cycling performances (2 A g^−1^) of VN/CFC SIBs.

The electrochemical performance of VN/CFC and VN was then systematically measured by assembling sodium-ion batteries. The cyclic voltammetry (CV) curves of the VN/CFC and VN-based sodium-ion batteries at 0.1 mV s^−1^ V (vs. Na/Na^+^) are shown in Figure 4a and Figure S4 with a voltage range from 0.01 V to 3 V. In the first cathodic scan (Figure 4a), the broad peak at around 1.03 V, which disappears in the subsequent cycle, is related to the generation of a solid electrolyte interface (SEI) film. In the anodic process, the peak at 0.549 V became sharp until the third and fourth cycles. For VN cells, similar to VN/CFC, there is a significant reduction peak at 1.05 V in the first cycle, which indicates the generation of an SEI film (Figure S4) [34]. In addition, there are two reduction peaks near 0.55 V and 0.39 V, which were not seen in the VN/CFC samples because the peaks of the carbon cloth were too strong. The nearly overlapping CV curves during the second and third cycles indicate the high reversibility of the VN electrode, but the response current is not significant. The reaction mechanism during the charge–discharge process can be summarized as follows:

VN + xNa^+^ + xe^−^ ↔ Na_x_VN.

The electrochemical cycling performance of the VN/CFC at a current density of 100 mA g^−1^ is shown in Figure 4b. The discharge–charge capacities of the VN/CFC-based battery were 583.7 mAh g^−1^ and 362.9 mAh g^−1^ during the initial cycle, which showed a large capacity decline with a coulombic efficiency (CE) of 62.17%. The capacity decline in the first cycle corresponds to the CV curve in Figure 4a, most likely due to the generation of SEI films [39]. After 100 cycles, the VN/CFC electrode exhibits a relatively high discharge capacity of 368.4 mAh g^−1^, compared with 97.2 mAh g^−1^ for VN, which may be due to the agglomeration of the material and the poor conductivity of the inactive binder. The VN electrode displays a discharge capacity of 260 mAh g^−1^ and a charge capacity of 108.0 mAh g^−1^ with a CE of 41.44% in the first cycle (Figure 4b). The galvanostatic discharge–charge curves of the VN/CFC SIBs electrodes at 0.1 A g^−1^ at the 1st, 2nd, 5th, 10th, 50th, and 100th cycles are shown in Figure 4c. As seen in the corresponding charge–discharge curve in Figure S5, after a large capacity loss in the first circle, the charge–discharge curve of the subsequent cycle has a higher degree of coincidence, but its capacity performance is worse than that of the VN/CFC sample. Figure S6 shows the cycle performance of CFC for SIBs and PIBs at 0.1 A g^−1^ current density. We can see that CFC, as a typical carbon material, also has a certain capacity for storing sodium/potassium when used alone as an anode. Notably, the VN/CFC electrode completely avoids the addition of a conductive agent and binder, reducing the capacity loss and further resulting in excellent electrochemical performances.

The rate capabilities of VN/CFC show outstanding average discharge-specific capacities of 433.3, 330.9, 299.6, 279.7, 236.9, and 174.9 mAh g^−1^ at various current densities of 0.1 0.2, 0.5, 1, 2, and 5 A g^−1^, respectively (Figure 4d). When the current density reverses from 5 to 0.1 A g^−1^, the discharge capacity of VN/CFC regained 350.2 mAh g^−1^. The excellent performance of VN/CFC-based SIBs proves the superiority of this structure. In addition, it was found that VN/CFC exhibits a relatively high capacity of 227 mAh g^−1^ after 1000 cycles at 2.0 A g^−1^, proving that the material can still have an excellent performance under a high current density for long-term cycling (Figure 4e).

**Figure 5:**
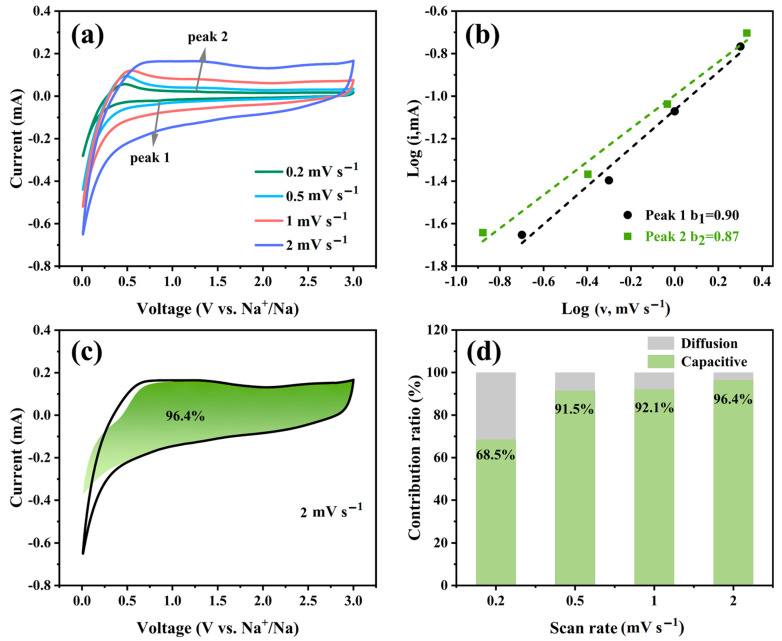
(**a**) CV curves (scan rate in 0.2~2 mV s^−1^), (**b**) function of *log i* with respect to *log v* at peak currents 1 and 2, (**c**) pseudo capacitance contribution obtained from a CV curve (2 mV s^−1^ scan rate), and (**d**) pseudo capacitance contribution (scan rate in 0.2~2 mV s^−1^) of the VN/CFC-based electrode.

The CV curves of VN/CFC-based SIBs were tested at different scanning rates (0.2, 0.5, 1, and 2 mV s^−1^) in the voltage range of 0.01~3 V to study their electrochemical kinetic properties (Figure 5). It can be seen from Figure 5a that there are similar shapes in all CV curves. In addition, Figure 5b shows that the b values of peak 1 and peak 2 are 0.90 and 0.87, respectively (calculated according to Formula (1) or (2), where a and b are variable parameters, *i* is the peak current, and *v* is the sweep rate), indicating that the electrochemical reaction is more controlled via pseudo capacitance [40]. The ratio of the capacitive contribution can be expressed by Formula (3) or (4) [41]. According to the calculations, the contribution rates of capacitance control are 68.5%, 91.5%, 92.1%, and 96.4% when the scanning rates are 0.2, 0.5, 1, and 2 mV s^−1^, respectively (Figure 5d). As the scanning rate increases, the contribution of capacitance control increases, which further demonstrates that the capacitive contribution plays a main role in the VN/CFC material. 

The morphology of the VN/CFC electrode after 10 cycles at the current density of 0.5 A g^−1^ was observed, as shown in Figure S7, revealing that the structure of VN/CFC is as same as the images before cycling, further indicating that the VN/CFC is reversible during harsh sodiation/desodiation processes. This shows that VN/CFC anode materials have outstanding structural stability and play an important role in improving the performance of SIBs. The EIS spectra of VN and VN/CFC before cycling are compared in Figure S8. The value of charge transfer resistances (R*_ct_*) for VN/CFC is about 920.3 Ω, which is lower than 4280 Ω of VN. It can be seen from this result that the performance of VN/CFC anode materials in sodium-ion batteries is better than that of VN because the overall conductivity of the composite material is improved after the introduction of conductive carbon materials.

**Figure 6:**
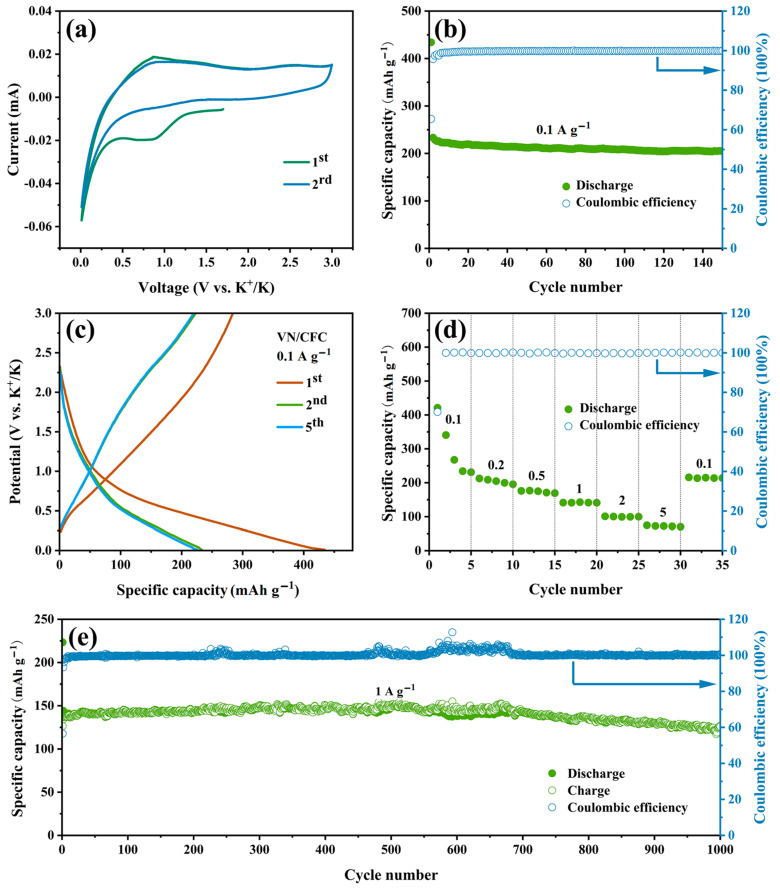
(**a**) CV curves (0.1 mV s^−1^), (**b**) cycling, (**c**) galvanostatic charge–discharge curves (0.1 A g^−1^), (**d**) rate, and (**e**) long life cycling performance (1 A g^−1^) of the VN/CFC anode in PIBs.

The VN/CFC and VN powder electrodes of the storage properties for the potassium-ion were investigated using CR2016 coin-type K-half-cells. As seen in the CV curves (scanning rate of 0.1 mV s^−1^ with a potential window from 0.01 to 3 V (vs. K/K^+^) of the VN/CFC electrode) in Figure 6a, a cathodic peak appeared near 0.72 V only in the first cycle, which could be explained by the formation of an SEI film [42]. In the oxidation process, there is a clear oxidation peak at 0.88 V. 

The VN/CFC-based PIB electrodes demonstrate a superior cycle capability at 0.1 A g^−1^ current density, as shown in Figure 6b. They achieved a discharge capacity of 434.2 mAh g^−1^ in the first cycle with an initial CE value of 65.44%. The low CE value is in good agreement with the previous analysis. The VN/CFC could deliver a stable capacity of 204.9 mAh g^−1^ after 150 cycles. The discharge–charge profiles of the VN/CFC electrode in the initial three cycles at a current density of 0.1 A g^−1^ are presented in Figure 6c. Although the formation of an SEI film caused a large capacity loss in the first cycle, the charge–discharge curves coincided with each other in the subsequent cycles, and the capacity remained stable.

The rate performance of the VN/CFC electrode in the potassium-ion battery was further evaluated at different current densities from 0.1 to 5 (Figure 6d). The average discharge specific capacities values could reach 298.9, 204.3, 173.6, 141.5, 100.0, and 72.3 mAh g^−1^ at the current densities of 0.1, 0.2, 0.5, 1, 2, and 5 A g^−1^, respectively. An excellent discharge capacity of 214.3 mA h g^−1^ could be recovered when the current density returned to 0.1 A g^−1^. An excellent cycling stability of 125.0 mAh g^−1^ was achieved after 1000 cycles of the VN/CFC electrode at a high current density of 1 A g^−1^. 

As a type of new vanadium-based anode material, these results for electrochemical performance highlight that the VN/CFC is not inferior to other reported vanadium-based sodium/potassium anode materials, which are shown in Table 1. Therefore, there is an excellent application prospect for VN/CFC materials in the field of energy storage.

## 4. Conclusions

In this study, we report on an efficient hydrothermal strategy to obtain a flexible self-supporting 3D electrode material VN/CFC. Compared with VN powders, the VN/CFC has a splendid electrochemical performance with SIB/LIB anodes. Using this material, additional conductive agents and binders, which could reduce the introduction of non-conductive components, can be avoided, thus reducing the capacity loss of active materials in the cycle process. In addition, this material benefits from the excellent electrical conductivity of the carbon fiber cloth, with evenly loaded VN and excellent pseudo capacitance characteristics of VN material. This unique support structure can alleviate the volume change in the cycle process and effectively prevent the aggregation of VN particles in the cycle process. In brief, a VN/CFC anode with an excellent Na/K storage performance has great potential in the application of flexible SIB/PIB anodes.

## Figures and Tables

**Table 1:** Electrochemical performance comparison of the VN/CFC with other V-based anode materials for SIBs/PIBs.

Sample	Fields	Current Density (A g^−1^)	Cycle Number	Capacity Retention (mAh g^−1^)
VN@CF [30]	SIBs	0.1	500	204
VNQD@NC HSs [32]	SIBs	1	1400	306
VN/CNFs [37]	SIBs	2	4000	237
VN@rGO [43]	SIBs	1	10,000	155
VN-QDs/CM [19]	PIBs	0.1	100	228
V_2_O_3_@PNCNFs [44]	PIBs	0.05	500	230
VS_4_/SnS@C [45]	PIBs	1	6000	168.4
FeVO_4_/C composite [46]	PIBs	0.3	2000	250
VN [47]	SIBs	0.2	100	156.1
VN/CFC	SIBs	0.1	100	368.4
		2	1000	227.0
	PIBs	0.1	150	204.9
		1	1000	125.0

## Data Availability

The data presented in this study are available on reasonable request from the corresponding author.

## Supplementary Materials

The following supporting information can be downloaded at: https://www.mdpi.com/article/10.3390/ma16175820/s1, Figure S1: Vanadium Nitride Nanoparticles Grown on Carbon Fiber Cloth as an Advanced Binder-Free Anode for the Storage of Sodium and Potassium Ions. Figure S2: SEM micrographs of (a,b) VN precursor, (c,d) VN, (e) HAADF images and corresponding elemental mapping distribution of VN. Figure S3: High-resolution O 1s spectra recorded for the VN/CFC composite material. Figure S4: CV curve of VN at a scan rate of 0.1 mV s^−1^ between 0.01 and 3 V. Figure S5: Galvanostatic charge-discharge curves of VN-based anodes at 0.1 A g^−1^ rate. Figure S6: Cycle performance of CFC for SIBs and PIBs at 0.1 A g^−1^ current density. Figure S7: The ex-situ SEM images of VN/CFC electrode after 10 cycles at a current density of 0.5 A g^−1^. Figure S8: Nyquist plots of VN powder and VN/CFC as the anode materials for SIBs before cycling.

## References

[1] Ipadeola A.K., Eid K., Abdullah A.M. (2023). Porous transition metal-based nanostructures as efficient cathodes for aluminium-air batteries. Curr. Opin. Electrochem..

[2] Bu Y., Wu Y., Li X., Pei Y. (2023). Operational risk analysis of a containerized lithium-ion battery energy storage system based on STPA and fuzzy evaluation. Process Saf. Environ. Prot..

[3] Abu S.M., Hannan M.A., Lipu M.S.H., Mannan M., Ker P.J., Hossain M.J., Mahlia T.M.I. (2023). State of the art of lithium-ion battery material potentials: An analytical evaluations. issues and future research directions. J. Clean. Prod..

[4] Ji L., Lin Z., Alcoutlabi M., Zhang X. (2011). Recent developments in nanostructured anode materials for rechargeable lithium-ion batteries. Energy Environ. Sci..

[5] Sharma R., Kumar H., Kumar G., Sharma S., Aneja R., Sharma A.K., Kumar R., Kumar P. (2023). Progress and challenges in electrochemical energy storage devices: Fabrication, electrode material, and economic aspects. Chem. Eng. J..

[6] Gao P., Yuan P., Yue T., Zhao X., Shen B. (2023). Recycling metal resources from various spent batteries to prepare electrode materials for energy storage: A critical review. J. Energy Storage.

[7] Nayak P.K., Yang L., Brehm W., Adelhelm P. (2018). From lithium-ion to sodium-ion batteries: Advantages, challenges, and surprises. Angew. Chem..

[8] Kim S.W., Seo D.H., Ma X.H., Ceder G., Kang K. (2012). Electrode Materials for Rechargeable Sodium-Ion Batteries: Potential Alternatives to Current Lithium-Ion Batteries. Adv. Energy. Mater..

[9] Myung J.Y., Myung S.T., Sun Y.K. (2018). Recent progress in rechargeable potassium batteries. Adv. Funct. Mater..

[10] Lei Y., Zhang J., Chen X., Min W., Wang R., Yan M., Xu J. (2022). From spent lithium-ion batteries to high performance sodium-ion batteries: A case study. Mater. Today Energy.

[11] Huang Z., Gu Z., Heng Y., Ang E.H., Geng H., Wu X. (2023). Advanced layered oxide cathodes for sodium/potassium-ion batteries: Development, challenges and prospects. Chem. Eng. J..

[12] Kei K., Mouad D., Tomooki H., Shinichi K., Shinichi K. (2018). Towards K-ion and Na-ion batteries as “beyond Li-ion”. Chem. Rec..

[13] Liu Q., Hu Z., Li W., Zou C., Jin H., Wang S., Chou S., Dou S.X. (2021). Sodium transition metal oxides: The preferred cathode choice for future sodium-ion batteries?. Energy Environ. Sci..

[14] Liu S. (2019). Na_2_Ru_0.8_Mn_0.2_O_3_: A novel cathode material for ultrafast sodium ion battery with large capacity and superlong cycle life. J. Power Sources.

[15] Cao Y., He Y., Gang H., Wu B., Yan L., Wei D., Wang H. (2023). Stability study of transition metal oxide electrode materials. J. Power Sources.

[16] Pan J., Li C., Peng Y., Wang L., Li B., Zheng G., Song M. (2023). Application of transition metal (Ni, Co and Zn) oxides based electrode materials for ion-batteries and supercapacitors. Int. J. Electrochem. Sci..

[17] Dong S., Chen X., Gu L., Zhou X., Xu H., Wang H., Liu Z., Han P., Yao J., Wang L. (2011). Facile preparation of mesoporous titanium nitride microspheres for electrochemical energy storage. ACS Appl. Mater. Interfaces.

[18] Yang H., Xu R., Yao Y., Zhou X.F., Yu Y. (2019). Multicore–Shell Bi@N-doped Carbon Nanospheres for High Power Density and Long Cycle Life Sodium- and Potassium-Ion Anodes. Adv. Funct. Mater..

[19] Wu H., Yu Q., Lao C.Y., Qin M., Wang W.A., Liu Z., Man C., Wang L., Jia B., Qu X. (2019). Scalable synthesis of VN quantum dots encapsulated in ultralarge pillared N-doped mesoporous carbon microsheets for superior potassium storage. Energy Storage Mater..

[20] Wang J., Yue X., Xie Z., Abudula A., Guan G. (2021). MOFs-derived transition metal sulfide composites for advanced sodium ion batteries. Energy Storage Mater..

[21] Lim Y.V., Li X.L., Yang H.Y. (2021). Recent Tactics and Advances in the application of metal sulfides as high-performance anode materials for rechargeable sodium-ion batteries. Adv. Funct. Mater..

[22] Xie J.M., Zhuang R., Du Y.X., Pei Y.W., Tan D.M., Xu F. (2023). Advances in sulfur-doped carbon materials for use as anodes in sodium-ion batteries. New Carbon Mater..

[23] Li Z., Gao Y., Huang H., Wang W., Zhang J., Yu Q. (2023). Development of electrode materials for flexible potassium-ion batteries. Compos. Part B Eng..

[24] Huang Y., Haider R., Xu S., Liu K., Ma Z.-F., Yuan X. (2022). Recent Progress of Novel Non-Carbon Anode Materials for Potassium-Ion Battery. Energy Storage Mater..

[25] Xu L., Chen X., Guo W., Zeng L., Yang T., Xiong P., Chen Q., Zhang J., Wei M., Qian Q. (2021). Co-construction of sulfur vacancies and carbon confinement in V_5_S_8_/CNFs to induce an ultra-stable performance for half/full sodium-ion and potassium-ion batteries. Nanoscale.

[26] Xu L., Guo W., Zeng L., Xia X., Wang Y., Xiong P., Chen Q., Zhang J., Wei M., Qian Q. (2021). V_3_Se_4_ embedded within N/P co-doped carbon fibers for sodium/potassium ion batteries. Chem. Eng. J..

[27] Kim H., Kim J.C., Bianchini M., Seo D.H., Rodriguez-Garcia J., Ceder G. (2017). Recent progress and perspective in electrode materials for K-ion batteries. Adv. Energy Mater..

[28] Yang J., Zhou X., Wu D., Zhao X., Zhou Z. (2017). S-doped N-rich carbon nanosheets with expanded interlayer distance as anode materials for sodium-ion batteries. Adv. Mater..

[29] Wang W., Bao J.Z., Sun C.F. (2020). Liquid-phase exfoliated WS_2_-graphene composite anodes for potassium-ion batteries. Chin. J. Struct. Chem..

[30] Liu R., Yang L., Wang W., Zhao E., Wang B., Zhang X., Liu H., Zeng C. (2023). Surface redox pseudocapacitance-based vanadium nitride nanoparticles toward a long-cycling sodium-ion battery. Mater. Today Energy.

[31] Peng Q., Rehman J., Eid K., Alofi A.S., Laref A., Albaqami M.D., Alotabi R.G., Shibl M.F. (2022). Vanadium Carbide (V_4_C_3_) MXene as an Efficient Anode for Li-Ion and Na-Ion Batteries. Nanomaterials.

[32] Yuan J., Hu X., Chen J.X., Liu Y.J., Huang T.Z., Wen Z.H. (2019). In situ formation of vanadium nitride quantum dots on N- doped carbon hollow spheres for superior lithium and sodium storage. J. Mater. Chem. A.

[33] Cheng H., García-Aráez N., Hector A.L. (2020). Synthesis of vanadium nitride–hard carbon composites from cellulose and their performance for sodium-ion batteries. ACS Appl. Energy Mater..

[34] Zhang L., Wang W.A., Lu S., Xiang Y. (2021). Carbon anode materials: A detailed comparison between Na-ion and K-ion batteries. Adv. Energy Mater..

[35] Chao D., Zhu C., Yang P., Xia X., Liu J., Wang J., Fan X., Savilov S.V., Lin J., Fan H.J. (2016). Array of nanosheets render ultrafast and high-capacity Na-ion storage by tunable pseudocapacitance. Nat. Commun..

[36] Cao D., Kang W., Wang W., Sun K., Wang Y., Ma P., Sun D. (2020). Okra-Like Fe_7_S_8_/C@ZnS/N-C@C with Core–Double-Shelled Structures as Robust and High-Rate Sodium Anode. Small.

[37] Xu L., Xiong P., Zeng L., Liu R., Liu J., Luo F., Li X., Chen Q., Wei M., Qian Q. (2020). Facile fabrication of a vanadium nitride/carbon fiber composite for half/full sodium-ion and potassium-ion batteries with long-term cycling performance. Nanoscale.

[38] Zeng F., Lu T., He W., Chu S., Qu Y., Pan Y. (2021). In situ carbon encapsulation of ultrafine VN in yolk-shell nanospheres for highly reversible sodium storage. Carbon.

[39] Cheng Q., Deng Q., Zhong W., Tan T., Liu X., Chen C., Hu J., Lin Z., Huang K., Yang C. (2023). Criticality of solid electrolyte interphase in achieving high performance of sodium-ion batteries. Chem. Eng. J..

[40] Liang Y., Song N., Zhang Z., Chen W., Feng J., Xi B., Xiong S. (2022). Integrating Bi@C Nanospheres in Porous Hard Carbon Frameworks for Ultrafast Sodium Storage. Adv. Mater..

[41] Chen H., Sun N., Zhu Q., Soomro R.A., Xu B. (2022). Microcrystalline Hybridization Enhanced Coal-Based Carbon Anode for Advanced Sodium-Ion Batteries. Adv. Sci..

[42] Huang Y., Ding S., Xu S., Ma Z.-F., Wang J., Yuan X. (2022). Highly effective solid electrolyte interface on SnO_2_@C enabling stable potassium storage performance. Chem. Eng. J..

[43] Wei S., Wang C., Chen S., Zhang P., Zhu K., Wu C., Song P., Wen W., Song L. (2020). Dial the Mechanism Switch of VN from Conversion to Intercalation toward Long Cycling Sodium-Ion Battery. Adv. Energy Mater..

[44] Jin T., Li H., Li Y., Jiao L., Chen J. (2018). Intercalation pseudocapacitance in flexible and self-standing V_2_O_3_ porous nanofibers for high-rate and ultra-stable K ion storage. Nano Energy.

[45] Cao L., Luo B., Xu B., Zhang J., Wang C., Xiao Z., Li S., Li Y., Zhang B., Zou G. (2021). Stabilizing Intermediate Phases via Efficient Entrapment Effects of Layered VS_4_/SnS@C Heterostructure for Ultralong Lifespan Potassium-Ion Batteries. Adv. Funct. Mater..

[46] Niu X., Zhang Y., Tan L., Yang Z., Yang J., Liu T., Zeng L., Zhu Y., Guo L. (2019). Amorphous FeVO_4_ as a promising anode material for potassium-ion batteries. Energy Storage Mater..

[47] Hu T., Yang W., Wang C., Bu Y., Jin F., Zhang D., Gu M., Liu W., Liang Q., Liu R. (2021). Multilayer Porous Vanadium Nitride Microsheets Anodes for Highly Stable Na-ion Batteries. Chem. Res. Chin. Univ..

