# Supplementary material for: Vanadium Nitride Nanoparticles Grown on Carbon Fiber Cloth as an Advanced Binder-Free Anode for the Storage of Sodium and Potassium Ions
Source: Materials (Basel). 2023 Aug 25;16(17):5820. doi: 10.3390/ma16175820 (PMC10488474; doi:10.3390/ma16175820)
Supplement: Supplementary file 1 [file materials-16-05820-s001.zip › materials-2546931-supplementary.pdf]

Supporting information

Vanadium Nitride Nanoparticles Grown on Carbon Fiber Cloth as an Advanced Binder-Free Anode for the Storage of Sodium and Potassium Ions

Yiwei Qin[1], Haimin Zhang[2], Yanghe Jiachen[3], Jing Yang[3], Wei Li[4], Xiaojun Zhao[1*], Sainan Liu[3]*

[1]*School of Materials Science and Engineering, Central South University, Changsha 410083, China; yiweiqin.csu@foxmail.com*

[2]*Hunan Zoomlion Neo Material Technology Co., Ltd., Changsha 410083, China; zhanghaimin@csu.edu.cn*

[3]*School of Minerals Processing and Bioengineering, Central South University, Changsha 410083, China; 8204211522@csu.edu.cn (Y.J.); 215612104@csu.edu.cn (J.Y.)*

[4]*Powder Metallurgy Research Institute, Central South University, Changsha 410083, China; csuliw@csu.edu.cn*

***Correspondence:** *zhaoxj@csu.edu.cn (X.Z.); lsn@csu.edu.cn (S.L.)*

LIST OF CONTENTS

Fig. S1. Comparation of SEM morphology of (a, b) commercial and (c, d) treated carbon.

Fig. S2. SEM micrographs of (a, b) VN powder precursor, (c, d) VN powder, (e) HAADF images and corresponding elemental mapping distribution of VN.

Fig. S3. High-resolution O 1s spectra recorded for the VN/CFC composite material.

Fig. S4. CV curve of VN powder anode at a scan rate of 0.1 mV s^{-1} between 0.01 and 3 V.

Fig. S5. Galvanostatic charge-discharge curves of VN-based anodes at 0.1 A g^{-1} rate.

Fig. S6. Cycle performance of CFC for SIBs and PIBs at 0.1 A g^{-1} current density.

Fig. S7. The ex-situ SEM images of VN/CFC electrode after 10 cycles at a current density of 0.5 A g^{-1}.

Fig. S8. Nyquist plots of VN powder and VN/CFC as the anode materials for SIBs before cycling.

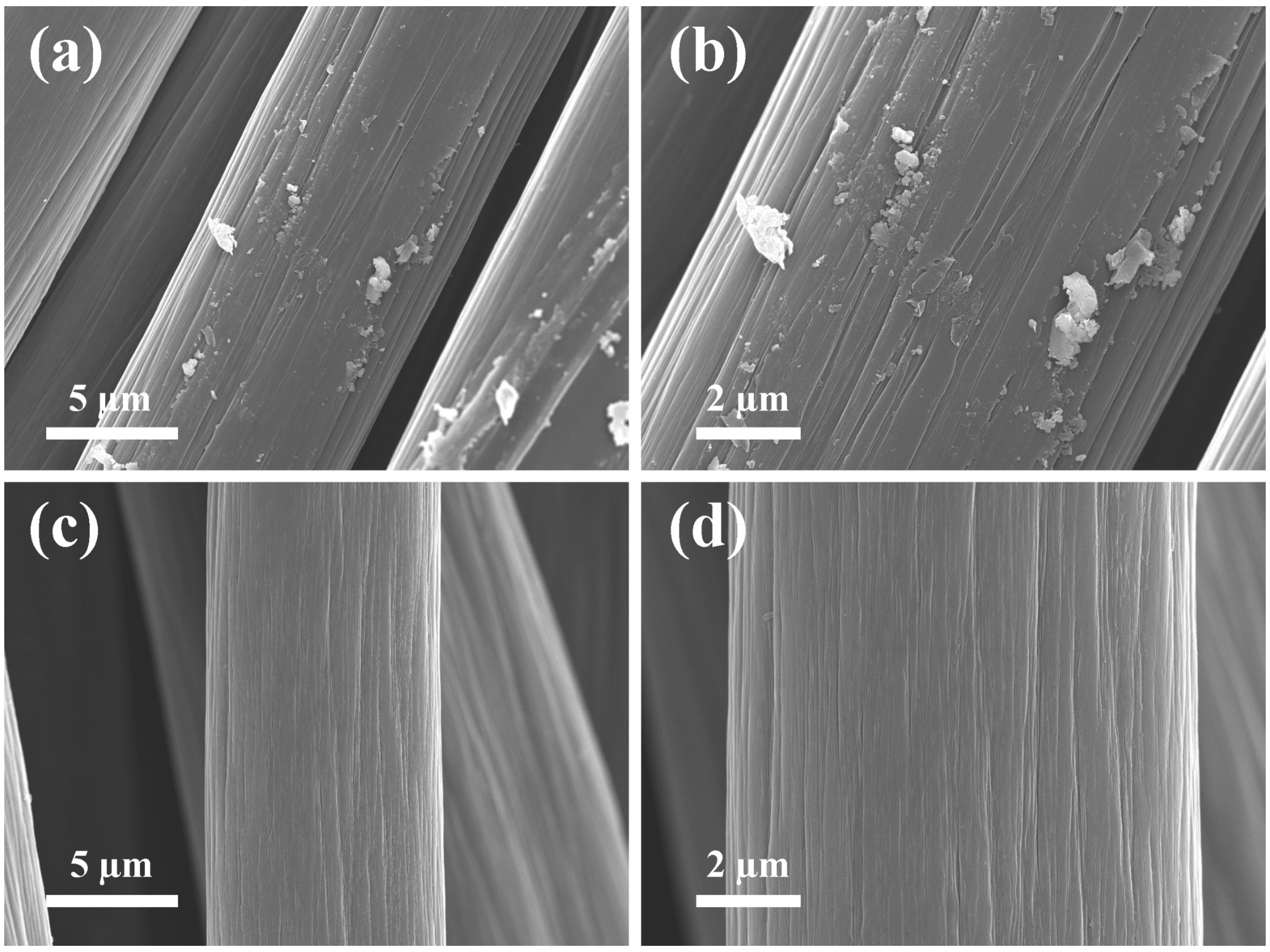

Fig. S1. Comparation of SEM morphology of (a, b) commercial and (c, d) treated carbon cloth.

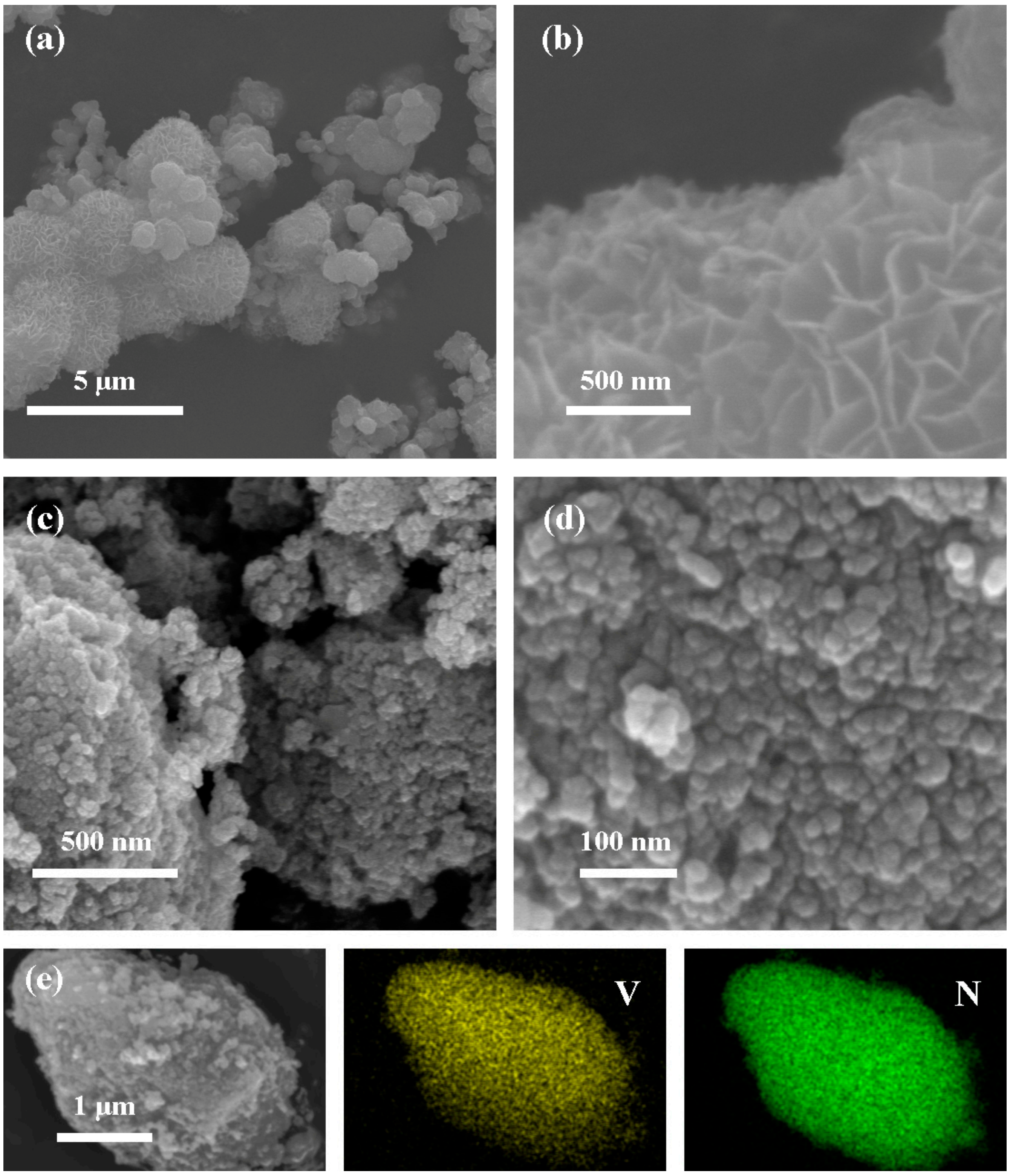

Fig. S2. SEM micrographs of (a, b) VN precursor, (c, d) VN, (e) HAADF images and corresponding elemental mapping distribution of VN.

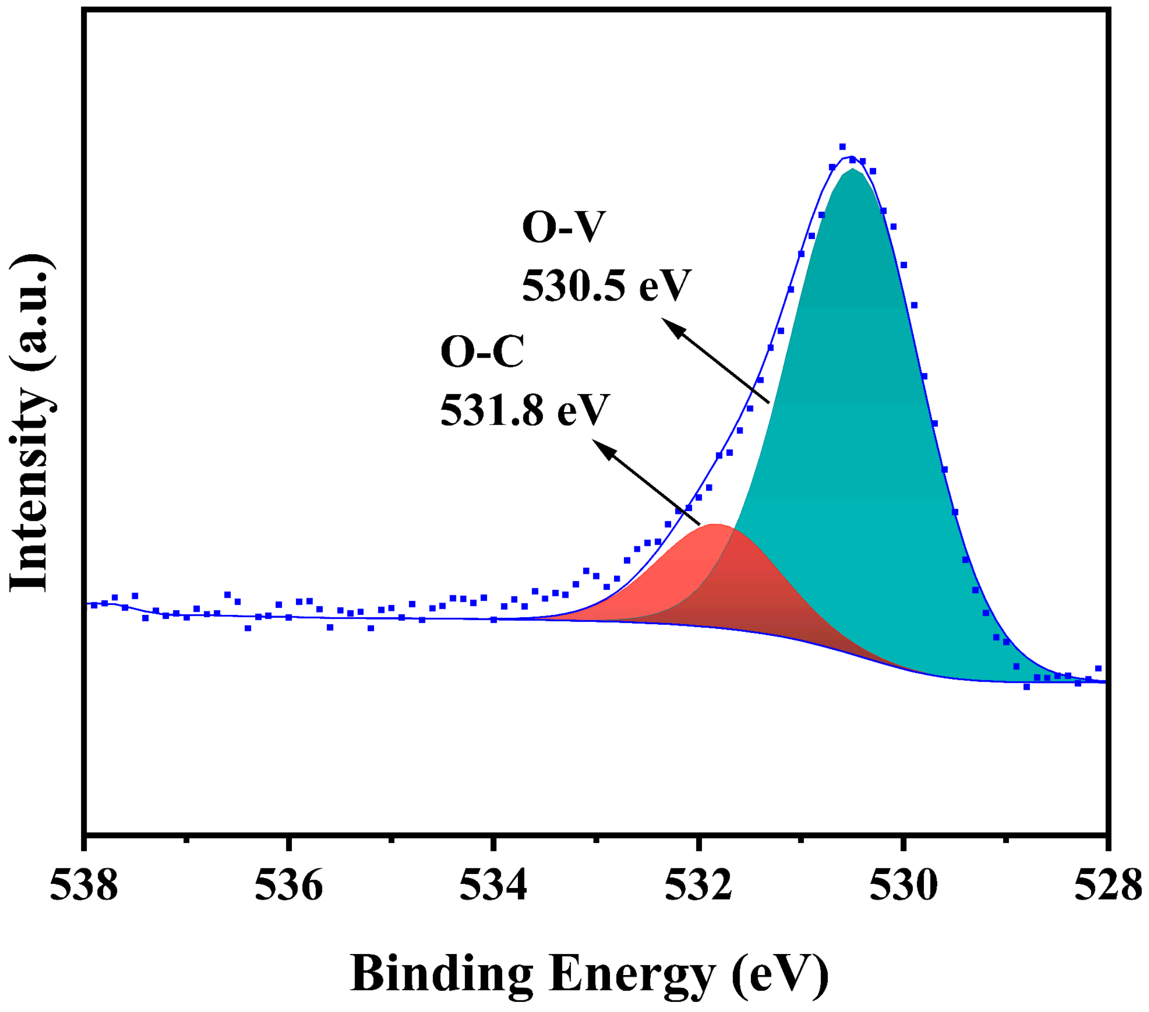

Fig. S3. High-resolution O 1s spectra recorded for the VN/CFC composite material.

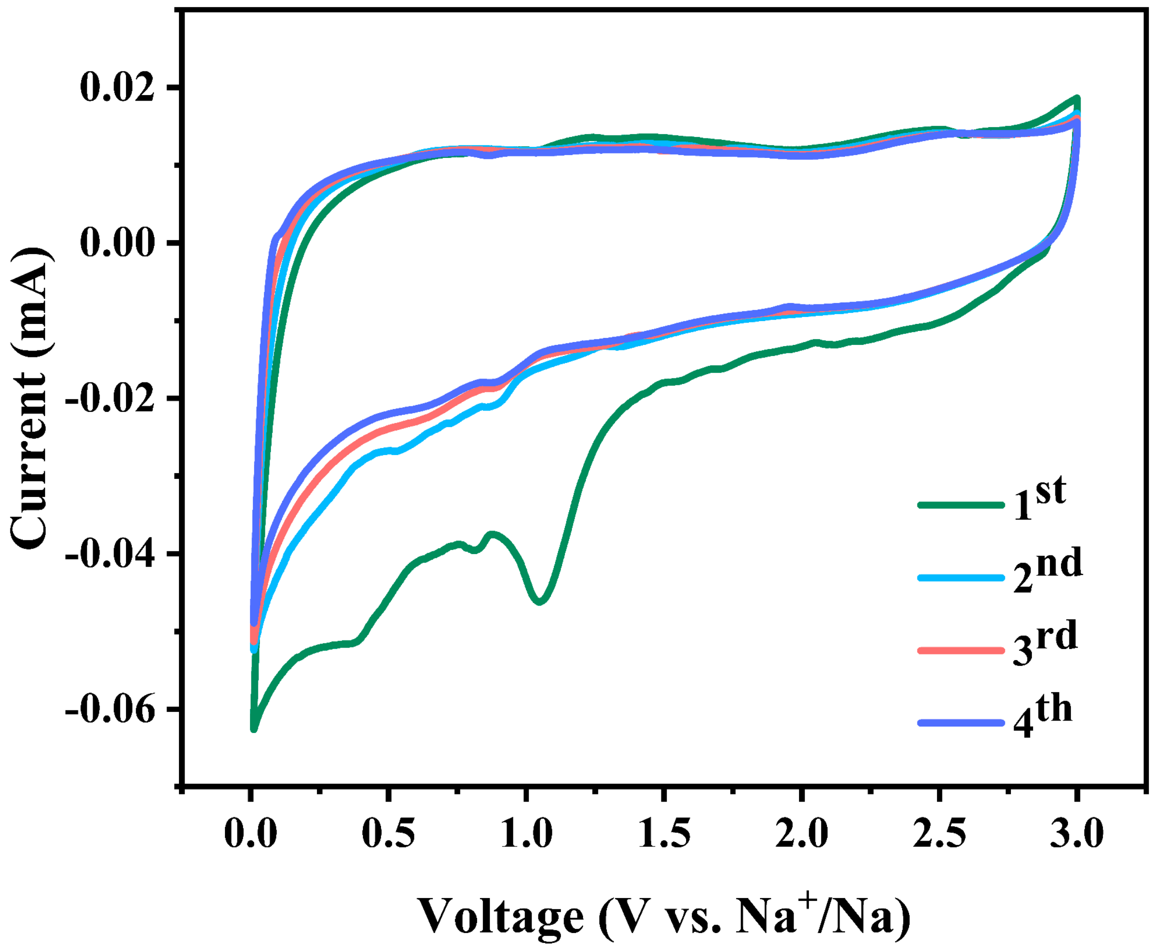

Fig. S4. CV curve of VN at a scan rate of 0.1 mV s^{-1} between 0.01 and 3 V.

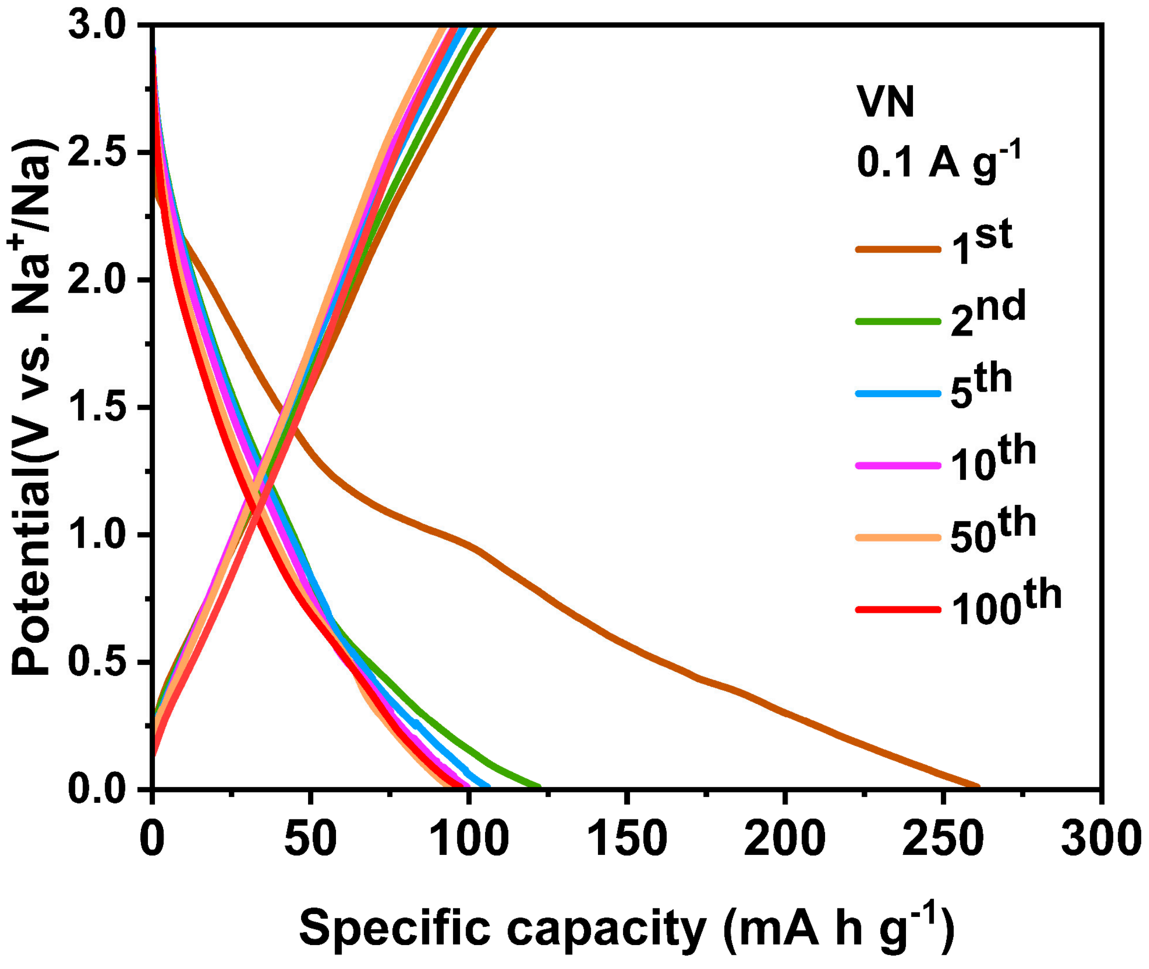

Fig. S5. Galvanostatic charge-discharge curves of VN-based anodes at 0.1 A g^{-1} rate.

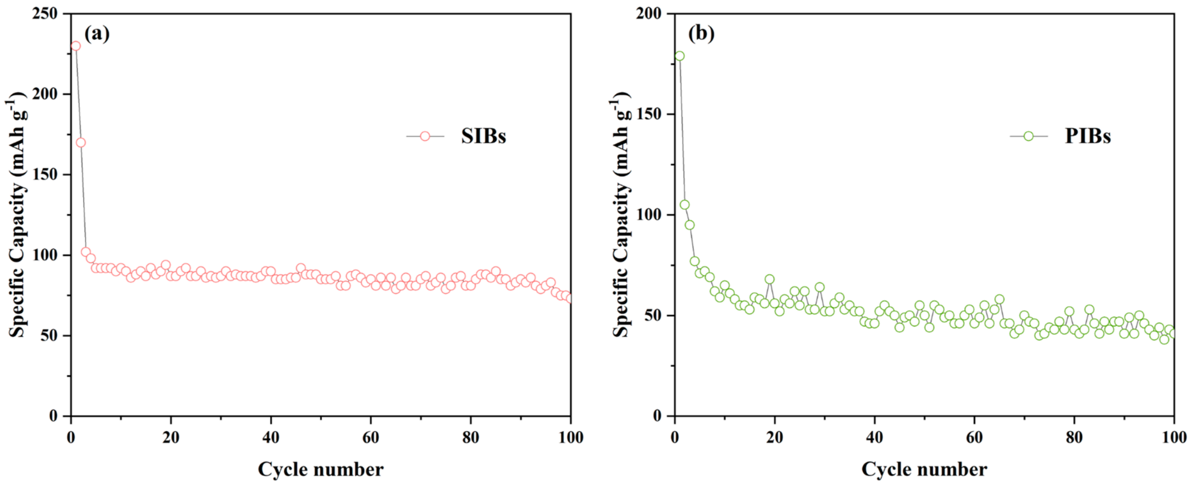

Fig. S6. Cycle performance of CFC for SIBs and PIBs at 0.1 A g^{-1} current density.

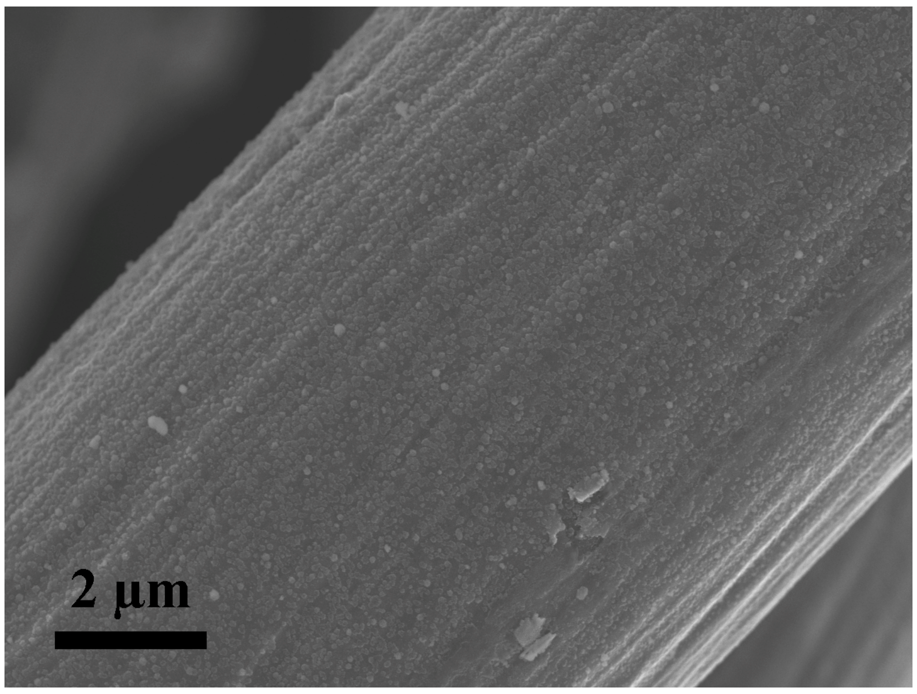

Fig. S7. The ex-situ SEM images of VN/CFC electrode after 10 cycles at a current density of 0.5 A g^{-1}.

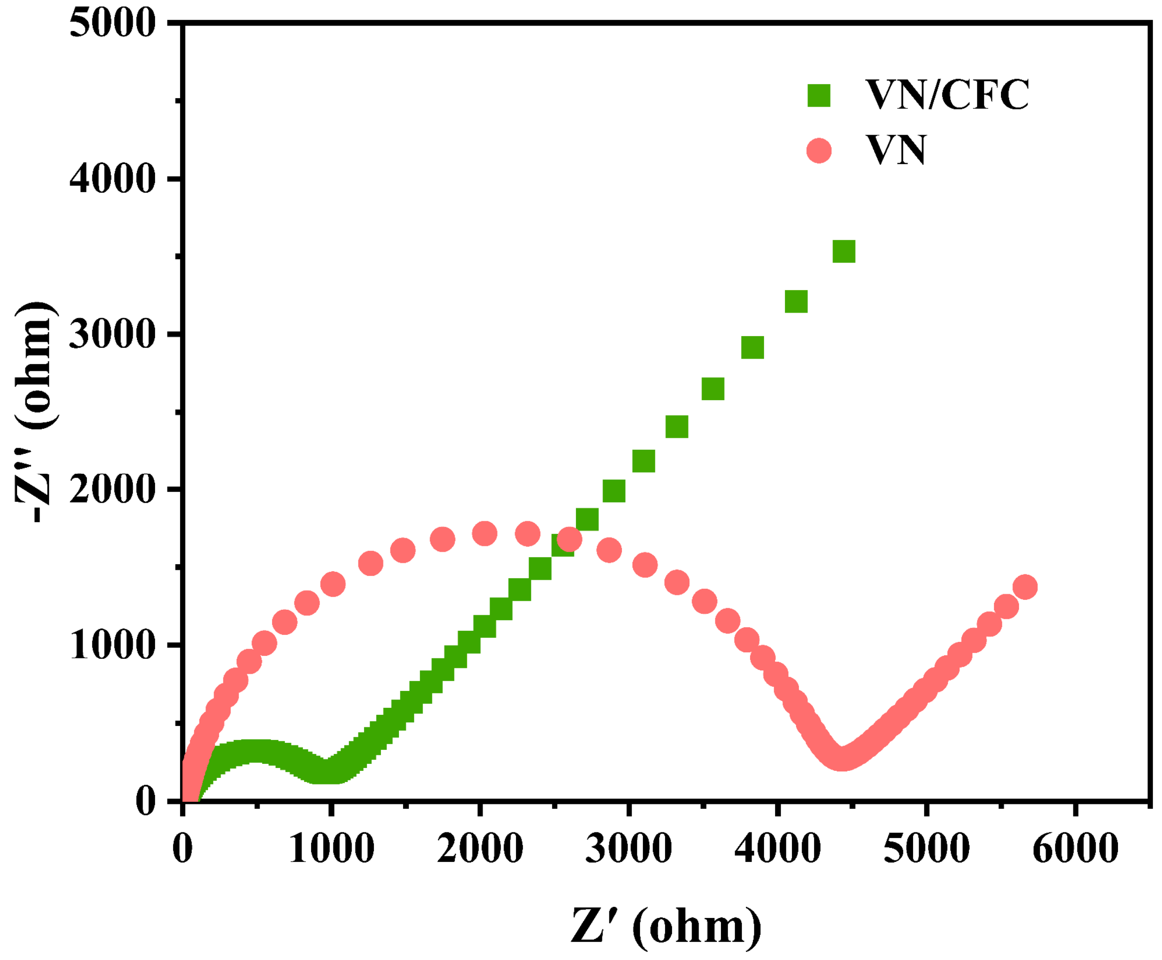

Fig. S8. Nyquist plots of VN and VN/CFC as the anode materials for SIBs before cycling.
